# Surface Plasmon Resonance Sensor Based on Polymer Photonic Crystal Fibers with Metal Nanolayers

**DOI:** 10.3390/s130100956

**Published:** 2013-01-15

**Authors:** Ying Lu, Cong-Jing Hao, Bao-Qun Wu, Mayilamu Musideke, Liang-Cheng Duan, Wu-Qi Wen, Jian-Quan Yao

**Affiliations:** Key Laboratory of Opto-electronics Information Technology (Ministry of Education), College of Precision Instrument and Opto-electronics Engineering, Tianjin University, Tianjin 300072, China; E-Mails: cathyhcj@163.com (C.-J.H.); baoqunwu@126.com (B.-Q.W.); mahira_laser@yahoo.cn (M.M.); duan072@tom.com (L.-C.D.); lasertju@163.com (W.-Q.W); jqyao@tju.edu.cn (J.-Q.Y.)

**Keywords:** polymer photonic crystal fiber, surface plasmon resonance sensor

## Abstract

A large-mode-area polymer photonic crystal fiber made of polymethyl methacrylate with the cladding having only one layer of air holes near the edge of the fiber is designed and proposed to be used in surface plasmon resonance sensors. In such sensor, a nanoscale metal film and analyte can be deposited on the outer side of the fiber instead of coating or filling in the holes of the conventional PCF, which make the real time detection with high sensitivity easily to realize. Moreover, it is relatively stable to changes of the amount and the diameter of air holes, which is very beneficial for sensor fabrication and sensing applications. Numerical simulation results show that under the conditions of the similar spectral and intensity sensitivity of 8.3 × 10^−5^–9.4 × 10^−5^ *RIU*, the confinement loss can be increased dramatically.

## 1. Introduction

Surface Plasmon Resonance (SPR) is a powerful direct sensing technique. SPR-based sensing methods are currently considered to be the most advanced real-time and label-free detection technology. Propagating at the metal–dielectric interface, surface plasmons are extremely sensitive to changes in the refractive index of the dielectric medium. This feature constitutes the core of many SPR sensors [1]. Surface plasmons resonance is one of the most promising optical techniques which refers to the excitation of surface plasmon polaritons, and it has broad applications in biology, environment, chemistry, medicine, *etc.* [2–5].

In recent years, there have been many investigators interested in designing photonic crystal fiber (PCF)-based SPR sensors. Their sensing mechanism is though coupling the leaky core mode to the plasmon to achieve resonance sensing. The use of the photonic crystal fiber, with its flexible design, makes it easy to equate the effective index of the core mode to that of the material under test. Thus the phase matching condition between the core mode and the plasmon can be easily achieved at the required wavelength and when the phase matching condition is met, surface plasmons can be excited by light [6–9]. Especially important, the fabrication of sensors doesn't require removing the cladding or tapering of the fibers as traditional fibers do, so for the sensor packaging, there is no problem. The PCF-based SPR sensing has huge potential application in the field of detection of biochemical substances. In recent years, PCF-based SPR sensors have attracted much attention for use in refractive index sensing of aqueous environments [10,11]. In 2006 Hassani proposed PCF-based SPR sensors and optimized micro-fluid design concepts, and showed that refractive index resolution of 10^−4^ *RIU* could be achieved in this structure [12]. In 2008, Hautakorpi proposed and numerically analyzed a three-hole photonic crystal fiber SPR sensor, with a gold film deposited on the inner wall of the three holes. Numerical results indicated that the optical loss of the Gaussian guided mode can be made very small and that the refractive-index resolution for aqueous analytes is 1 × 10^−4^ *RIU* [10].

However, either coating the holes of a photonic crystal fiber with metal film or filling them with liquid sampled is difficult and time-consuming work. In this paper, we design a simple kind of polymer PCF with the cladding having only one layer of air holes near the edge of the fiber, and use it in SPR sensors after silver film is deposited on its outer side. Polymer PCFs have attracted much attention because of their unique characteristics such as flexibility, relatively large numerical aperture (NA) and low cost in coupling, easy termination and handling, relatively high resistance to fracture and perfect biocompatibility. These properties of polymer optical fiber make it very useful for a variety of optical sensing applications in biomedical fields [13].

In this work, we consider polymethyl methacrylate (PMMA) as the PCF material because it allows easy binding of biomolecules [14] and can be routinely fabricated with a wealth of different structures [15]. PMMA has been used for PCF with other advantages such as being the most transparent polymer, low-cost, widely available and so on [16]. Although the polymer optical fiber has much larger losses than common silica fiber, and may be not suitable for long-distance signal transmission, it is quite useful for sensing applications [17].

As the mode area of the fiber designed in this paper is very large, the light field can be easily limited within the core region, even though only one layer of air holes is available, leading to very low losses of the fiber. It is also very convenient to connect and couple this fiber with the light source outside.

In this paper, surface plasmon resonance sensors based on this kind of photonic crystal fiber have been analyzed though the finite element method (FEM), regularity of the resonant wavelength changing with refractive index of the sample has been numerically simulated, and resonant wavelength detection as well as intensity detection sensitivity have also been discussed. Numerical simulation results show that both spectral and intensity sensitivity are in the range of 8.3 × 10^−5^–9.4 × 10^−5^ *RIU*. In order to increase the transmission loss appropriately, and improve the sensitivity of the sensor, we have decreased the diameter of the fiber core to 125 μm and introduced more air holes in the core of the PCF, the calculation results show that the improved structure obtains higher transmission losses with similar sensitivity. Especially, both the fabrication of the sensor and the detection process are expected to be simplified.

## 2. Numerical Simulation and Analysis

### 2.1. PMMA Photonic Crystal Fiber-Based of 250 μm Diameter SPR Sensor

**Figure 1.**
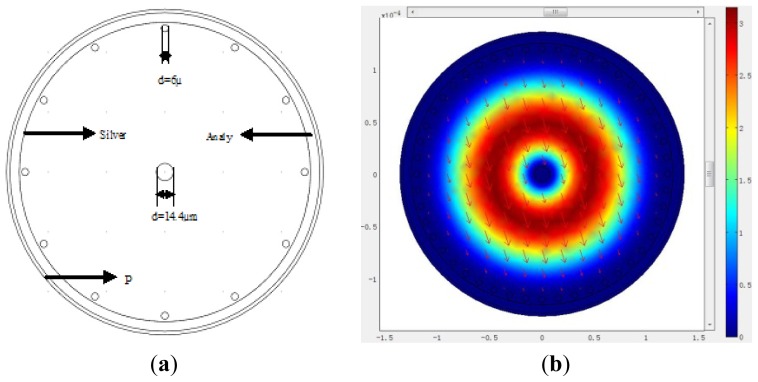
(**a**) Structure diagram of the large-mode-area plastic photonic crystal fiber; (**b**) Optical field distribution of the fundamental mode.

The mechanism of the PCF-based SPR is shown in Figure 1(a). This kind of photonic crystal fiber made of PMMA, whose structure design is based on [18,19], has only one layer of air holes (6 μm in diameter) near the edge of the fiber and one single hole (14.4 μm in diameter) in the core region, and the whole fiber diameter is 250 μm. We chose PMMA as the background material of polymer PCF because biomolecules can be attached directly to the surface of the holes of the fiber [14], and in a PMMA PCF, the thickness of the sensor layer can be reduced to 10 nm (the thickness of the antigen layer only) compared to a silica microstructured optical fiber (MOF) for a biosensor, since intermediate functionalization layers can be avoided. Markos *et al.* calculated the sensitivity for both thicknesses of ts = 40 nm and ts = 10 nm and they found the difference to be small [20]. In this paper, we choose the thickness of the film ts = 40 nm.

Silver film of 40 nm thickness is deposited on the outer side of the fiber, which is surrounded by an aqueous sample, which can reduce the refractive index contrast between cladding-core, thus enabling single-mode operation of the fiber above 500 nm [20]. The FEM with a perfectly matched layer (PML) is chosen to calculate the effective indices of fiber modes supported by the sensor, and more accurate confinement loss of the fiber can be obtained. Figure 1(b) represents field distribution of the fundamental mode. Refractive index of the PMMA fiber can be determined by the Sellmeier equation: n = C_1_ + C_2_λ^2^ + C_3_λ^−2^ + C_4_λ^−4^ + C_5_λ^−6^ + C_6_λ^−8^, where C_1_ = 2.399964, C_2_ = −8.308636E−2, C_3_ = −1.919569E−1, C_4_ = 8.720608E−2, C_5_ = −1.666411E−2, C_6_ = 1.169519E−3 [21]. The relative permittivity of silver (or refractive index) is obtained from an optical handbook [22].

**Figure 2.**
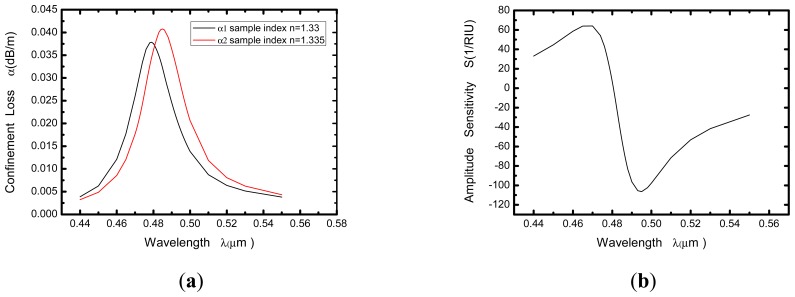
(**a**) Relationship between wavelength and attenuation constant of the fundamental mode of the hollow-core large-mode-area PCF. The black and red curves represent the refractive indices of the samples are 1.33 and 1.335 (Δλpeak ≈ 6 nm), respectively; (**b**) Intensity detection sensitivity curve.

As shown in Figure 2(a), a relationship between wavelength and confinement loss constant of the fundamental mode is obtained, in which the black and red curves representing the refractive indices of the samples are 1.33 and 1.335, respectively. This diagram presents that a sharp loss peak in the range of 470–490 nm for each curve. That is because the resonance between the core mode and the surface plasmon makes great energy loss of light field in the core.

The attenuation constant of the fundamental mode is calculated for different incident light wavelengths. The wavelength with maximal transmission loss can be identified. It is easy to prove that the optical fiber transmission loss coefficient *α*_loss_ is:

(1) αloss=10lge⋅α=10lge⋅2k0Im(neff)=8.686⋅2πλ⋅Im[neff](dB/m)

where *k*_0_ is the wave number (*k*_0_=2π/*λ*),*n_eff_* align is the mode effective refractive index.

When the refractive index of the sample changes from 1.33 to 1.335 (Δn*_a_* = 0.005), the resonance peak shifts toward longer wavelength, and the amount of shift is about 6 nm (Δ*λ*_peak_). If the instrumental peak-wavelength resolution is assumed to be Δ*λ*_min_ = 0.1 nm, the refractive index resolution of the corresponding sensor can be obtained as:

(2) $$\text{R}=\Delta n_{a}\Delta \lambda_{\min}/\Delta \lambda_{\text{peak}}=8.33\times 10^{-5}\text{RIU}$$

Another detection method is known as the power detection at a fixed single wavelength, which is also known as amplitude-based detection method. Assume that the wavelength of the light is *λ*, and the transmission length is L, and then the power detection sensitivity for the refractive index variation Δn*_a_* can be defined as:

(3) $$\text{S}(\lambda )=\left(P(L,\lambda ,n_{a}+\Delta n_{a})-P(L,\lambda ,n_{a})\right)/P(L,\lambda ,n_{a})/\Delta n_{a}$$

The sensor length L is typically limited by the modal transmission loss. A reasonable choice of a sensor length is L = 1/*α*(*λ*,n*_a_*). Such choice of a sensor length results in a simple definition of sensitivity for the small changes in the analyte refractive index [23]:

(4) $$\text{S}(\lambda )=1/P(L,\lambda ,n_{a})\cdot \partial P(L,\lambda ,n_{a})/\partial n_{a}=1/\alpha (\lambda ,n_{a})\cdot \partial \alpha (\lambda ,n_{a})/\partial n_{a}$$

In Figure 2(b) we present the amplitude sensitivity curve of the computed PCF-based SPR sensor. As can be seen in the diagram, the maximal sensitivity of about 106 *RIU*^−^*^1^* is achieved around 495 nm. It is typically a safe assumption that a 1% change in the transmitted intensity can be detected reliably, which leads to a sensor resolution of 9.43 × 10^−5^ *RIU*.

### 2.2. PMMA Photonic Crystal Fiber-Based of 125 μm Diameter SPR Sensor (Seven Air Holes)

**Figure 3.**
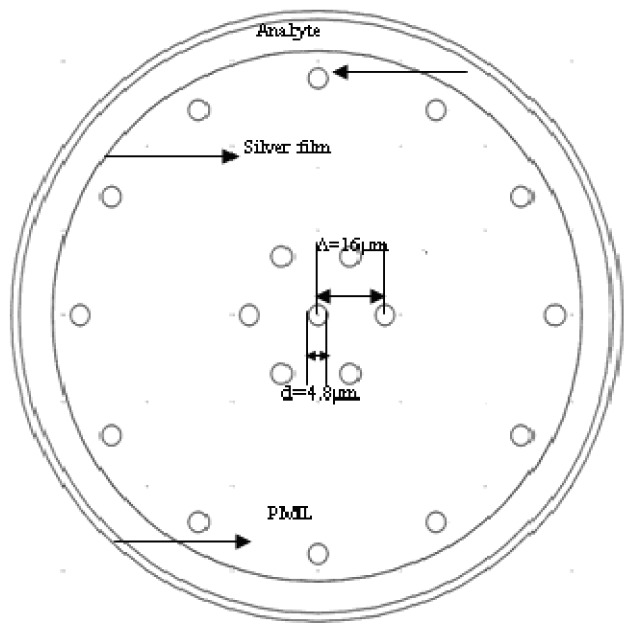
Structure diagram of the improved large-mode-area plastic PCF with 125 μm diameter and seven air holes.

An important parameter of the PMMA-based SPR sensor is the confinement loss. From the contents mentioned above, we can see that the confinement loss of the proposed 250 μm PMMA fiber is very low if the core of the fiber is large enough. Low-loss fiber may be very beneficial for signal transmission, but not always for sensing applications. In order to increase the transmission loss appropriately, and improve the sensitivity of the sensor, the diameter is decreased (125 μm) and air holes (six) are introduced in the core of the PCF, which is shown in Figure 3. The refractive index of a core-guided mode is lowered by doing so, and this can facilitate phase matching with a plasmon.

**Figure 4.**
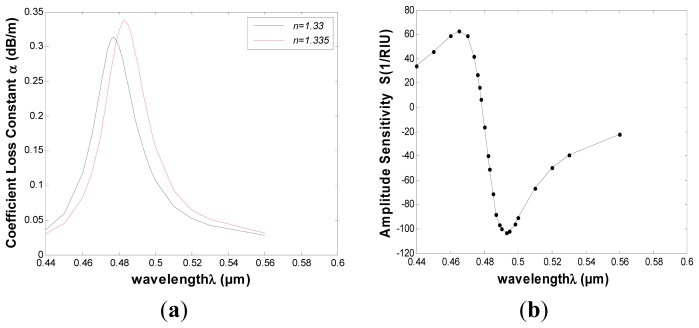
(**a**) Relationship between wavelength and attenuation constant of the fundamental mode of the PMMA PCF. The red and blue curves represent the refractive indices of the samples are 1.33 and 1.335 (Δ*λ*peak ≈ 6 nm), respectively; (**b**) Intensity detection sensitivity curve.

The simulated results of the improved PMMA-based SPR sensor are shown in Figure 4. The relationship between wavelength (*λ*) and attenuation constant of the fundamental mode (*α*) is obtained and shown in Figure 4(a) in which the black and red curves represent the refractive indices of the samples (1.33 and 1.335, respectively). When sample refractive index changes from 1.33 to 1.335 (Δn*_a_* = 0.005), the resonance peak shifts 6 nm (Δ*λ*_peak_) towards the longer wavelength, corresponding to the spectral detection resolution of 8.33 × 10^−5^ *RIU*. The maximal amplitude sensitivity is achieved at 493 nm and equals to 103 *RIU*^−1^, as shown in Figure 4(b). That a 1% change in the transmitted intensity can be detected reliably is also assumed, leading to a sensor resolution of 9.43 × 10^−5^ *RIU*.

Compared with the first structure it is clear that the fiber loss is greatly increased when six big air holes are added in the core region of the fiber and the diameter is decreased, while maintaining similar sensitivity. We conclude that increasing the air holes, or reducing the fiber diameter can further increase the confinement loss of the PMMA PCF.

### 2.3. Introduction of More Air Holes into the PMMA Photonic Crystal Fiber-Based of 125 μm Diameter SPR Sensor

**Figure 5.**
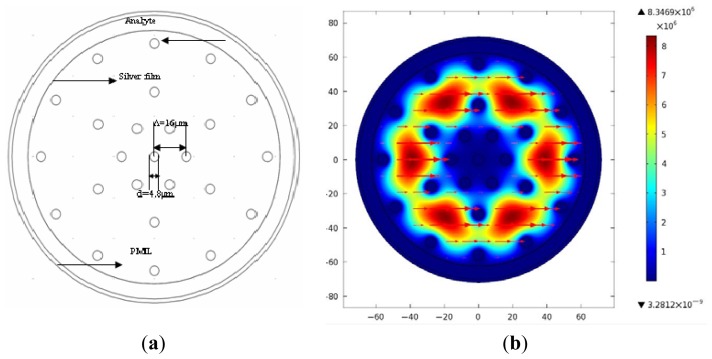
(**a**) the improved structure diagram of the large-mode-area plastic PCF with 125 μm diameter and more air holes; (**b**) Optical field distribution of the fundamental mode.

To improve the sensing properties, we add more air holes into the core of the PCF with the diameter of 125 μm, which is shown in Figure 5(a). The structure increases the transmission loss appropriately, and improves the sensitivity of the sensor. Figure 5(b) represents the fundamental mode field distribution, and the arrows indicate the polarization direction of the electric field.

**Figure 6.**
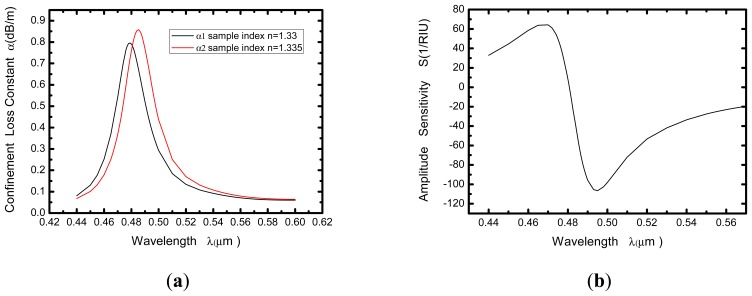
(**a**) Relationship between wavelength and attenuation constant of the fundamental mode of the PMMA PCF. The black and red curves represent the refractive indices of the samples are 1.33 and 1.335 (Δ*λ*_peak_ ≈ 6nm), respectively; (**b**) Intensity detection sensitivity curve.

The results of the improved PMMA-based SPR sensor are shown in Figure 6, which indicates that the fiber confinement loss is greatly increased again with the similar sensitivity. We can conclude that increasing the number of air holes with a smaller diameter can further increase the confinement loss of the PMMA PCF.

### 2.4. Optimizations of Structural Parameters

**Figure 7.**
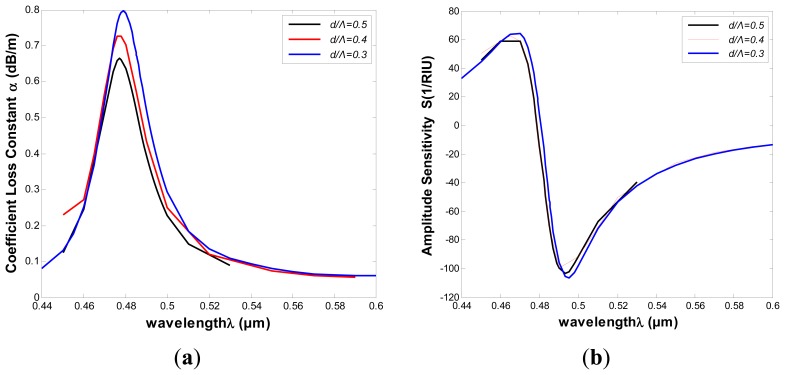
(**a**) The comparison of attenuation constant of the fundamental mode of different diameters of the air holes (**b**) The comparison of intensity detection sensitivity curves.

In order to optimize the structural parameters of the proposed PMMA PCF-based SPR sensor for high sensitivity, it is important to understand the effects these parameters have on the sensor properties. Considering the structure of Figure 5(a), the air holes of diameter *d*, has been employed to tune the phase matching conditions. In what follows, we will investigate the effect of size variation of the air holes. The diameter of the air holes was varied between 0.3Λ, 0.4Λ and 0.5Λ, while keeping all other structural parameters constant. Change in the confinement loss is shown in Figure 7(a) (n*_a_* = 1.33), which shows that the loss increased with the decreasing diameter of the air holes. However the sensitivity ultimately reaches the same value, as shown in Figure 7(b).

In Figure 7, we can observe that the amount of air holes plays a significant role in the confinement loss reduction, but the diameter of the air holes almost has no influence on the PMMA PCF-based SPR sensor. The curves show that it ultimately maintains the same properties of the fundamental mode at the resonant wavelength with the changed diameters of the air holes. This is different from silica fiber structures, which is sensitive to structure changes. These properties are beneficial to the fabrication of PMMA PCFs, and it are an advantage for a polymer PCF sensor.

**Figure 8.**
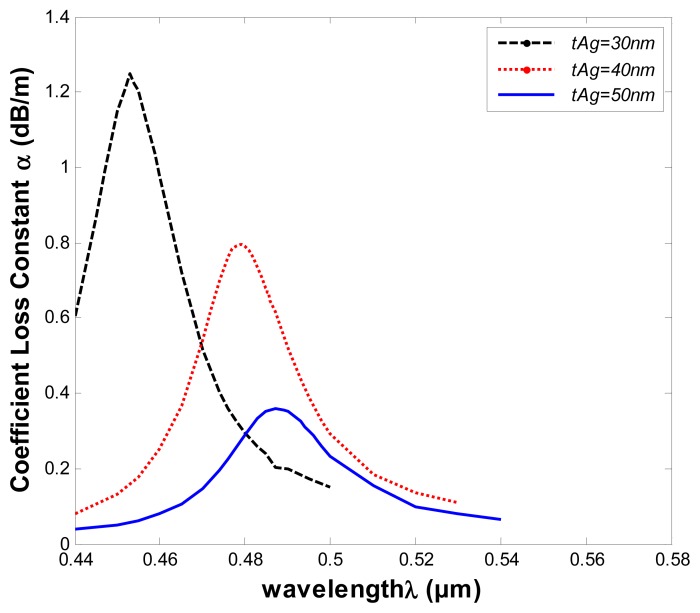
Loss spectra of the structure of Figure 5(a) at different silver layer thickness (t_Ag_ = 30 nm, 40 nm, 50 nm). Analyte refractive index (n*_a_* = 1.33).

Surface plasmon waves, like the surface excitations, are very sensitive to the thickness of the metallic layer, so we investigated the influence on sensing properties of changes of the thickness of silver t_Ag_, which is varied from 30 nm to 50 nm. As illustrated in Figure 8, the confinement loss spectra for the fundamental mode of the structure of Figure 5(a), in which the refractive index of the samples is 1.33 and other structural parameters are kept constant.

As shown in Figure 8, the resonant wavelength shifts to longer wavelengths as the thickness of the silver layer increases. The resonant wavelength shifts from about 1 nm to 2 nm for t_Ag_ values of 30 nm and 50 nm. In addition, the loss spectra shows a downward trend when the thickness of the silver layer increases. The electric field will be difficult to sense through the silver layer when the thickness is larger. Therefore, we should establish an appropriate t_Ag_ for the corresponding sensing regimes.

## 3. Conclusions

We have proposed a surface plasmon resonance sensor based on a polymer photonic crystal fiber coated with a silver film in an aqueous environment. Through the above calculations, the properties of the polymer photonic crystal fiber sensing are discussed in both the areas of resonant wavelength and intensity detection. Numerical results show that changes of the amount of air holes and air holes' diameter almost have no effect on sensitivity, which is very beneficial for sensor fabrication and sensing applications. It shows that excellent sensing characteristics of the structure can be achieved with both spectral and intensity sensitivity in the range of 8.3 × 10^−5^–9.4 × 10^−5^ *RIU*. Moreover, coating the holes of a PCF with metal film and filling of them with liquid sample are not needed, and both the coating and filling works can be done outside of the PCF, making it possible to achieve real-time, fast and convenient detection.
